# Differences in quality-of-life dimensions of Adult Strabismus Quality of Life and Amblyopia & Strabismus Questionnaires

**DOI:** 10.1007/s00417-017-3694-x

**Published:** 2017-05-29

**Authors:** Elizabeth S. van de Graaf, Gerard J. J. M. Borsboom, Geertje W. van der Sterre, Joost Felius, Huibert J. Simonsz, Henk Kelderman

**Affiliations:** 1 Department of Ophthalmology, Erasmus MC, University Medical Center Rotterdam, PO Box 2040, 3000 CA Rotterdam, The Netherlands; 2 Department of Public Health, Erasmus MC, University Medical Center Rotterdam, Rotterdam, The Netherlands; 3 Retina Foundation of the Southwest, Dallas, TX USA; 4 Department of Social Sciences, University of Leiden, Leiden, The Netherlands

**Keywords:** Quality of life, Adult strabismus, Questionnaires, Factor analysis, Clinical validation

## Abstract

**Purpose:**

The Adult Strabismus Quality of Life Questionnaire (AS-20) and the Amblyopia & Strabismus Questionnaire (A&SQ) both measure health-related quality of life in strabismus patients. We evaluated to what extent these instruments cover similar domains by identifying the underlying quality-of-life factors of the combined questionnaires.

**Methods:**

Participants were adults from a historic cohort with available orthoptic childhood data documenting strabismus and/or amblyopia. They had previously completed the A&SQ and were now asked to complete the AS-20. Factor analysis was performed on the correlation-matrix of the combined AS-20 and A&SQ data to identify common underlying factors. The identified factors were correlated with the clinical variables of angle of strabismus, degree of binocular vision, and visual acuity of the worse eye.

**Results:**

One hundred ten patients completed both questionnaires (mean age, 44 years; range, 38–51 years). Six factors were found that together explained 78% of the total variance. The factor structure was dominated by the first four factors. One factor contained psychosocial and social-contact items, and another factor depth-perception items from both questionnaires. A third factor contained seven items—only from the AS-20—on eye strain, stress, and difficulties with reading and with concentrating. A fourth factor contained seven items—only from the A&SQ—on fear of losing the better eye and visual disorientation, specific for amblyopia. Current visual acuity of the worse eye correlated with depth-perception items and vision-related items, whereas current binocular vision correlated with psychosocial and social-contact items, in 93 patients.

**Conclusions:**

Factor analysis suggests that the AS-20 and A&SQ measure a similar psychosocial quality-of-life domain. However, functional problems like avoidance of reading, difficulty in concentrating, eye stress, reading problems, inability to enjoy hobbies, and need for frequent breaks when reading are represented only in the AS-20. During the development of the A&SQ, asthenopia items were considered insufficiently specific for strabismus and were excluded a priori. The patients who generated the items for the AS-20 had, in majority, adulthood-onset strabismus and diplopia and were, hence, more likely to develop such complaints than our adult patients with childhood-onset strabismus and/or amblyopia.

## Introduction

It is widely accepted that amblyopia and strabismus have negative effects on quality of life, not only in children but also throughout adulthood [1]. The interest in identifying effective treatments for these conditions in adults requires that quality of life is taken into account in the evaluation of such treatments [2]. There are currently two condition-specific quality-of-life instruments available for this purpose: the Amblyopia and Strabismus Questionnaire (A&SQ) [3] and the Adult Strabismus Quality of Life Questionnaire (AS-20) [4, 5]. Although a few studies have used both questionnaires together in strabismus patients with and without amblyopia [6–8], not much attention has been given to the potential differences in applicability between the two instruments. It should be pointed out that these two questionnaires differ in important aspects, both in methods used for their development, and in the populations among which the instruments have been developed and validated.

The AS-20 was developed using two samples of adult patients who came for treatment with a median age of 39.5 years and of 51 years, respectively [4, 5]. This instrument was developed by Hatt et al. using an inductive method (grouping of descriptions): Thirty adult patients seeking medical attendance, the majority of whom reported diplopia, gave the input on 11 open-ended questions to generate 1301 quality-of-life phrases [4]. During the refinement phase with 29 patients, a pool of 181 phrases was reduced to 20 items and categorized by factor analysis into two subscales [5]. The A&SQ was developed by an expert focus group and validated among adults with childhood strabismus and/or amblyopia with a mean age of 36 years) [3]. The A&SQ was designed in 2002 by intuitive-deductive method [9] (logical categorization): Complaints of adults with childhood strabismus and /or amblyopia were gathered and categorized by a focus-group into themes and subsequently defined into domains of strabismus- and amblyopia-related quality of life [3]. The focus group consisted of one ophthalmologist, two orthoptists, two patients with childhood strabismus and amblyopia, and one statistician-methodologist. By factor analysis of the A&SQ, six factors were found that together explained 70.5% of the total variance [10]. The six factors overlapped with four out of the five pre-hypothesized A&SQ domains.

The present study aimed to compare the A&SQ and the AS-20 in terms of their qualitative coverage of quality-of-life domains. To this end, we performed factor analysis on a combined data set of AS-20 and A&SQ responses from the same individuals.

## Methods

### Study participants

We conducted a cross-sectional study. Respondents were a subgroup belonging to a larger cohort of 471 adult persons who were treated during childhood for amblyopia or strabismus. Their treatment took place between 1968 and 1974 at the orthoptic outpatient clinic of the Waterland Hospital in Purmerend, The Netherlands, and was provided by the single orthoptist in the Waterland area. During this time, the outpatient clinic served a large rural area with a very stable population. Thus, this historic cohort of adults may be regarded as a near-random sample of patients with amblyopia and strabismus. Of these 471 persons, 203 could be contacted 30–35 years after treatment. Of these 203 persons, 173 completed the A&SQ in 2002 [3], and 137 of these were orthoptically re-examined in 2003 [11, 12]. The 173 persons were contacted again for the purpose of the present study and were invited to complete the AS-20, which was sent to them, accompanied by a letter to inform about and obtain consent to participate in the study. The sample was differentiated into patients who had the condition of amblyopia only, strabismus only, or both conditions. Amblyopia was defined as Snellen visual acuity in the amblyopic eye of less than 0.5 at the beginning of childhood treatment, but the visual acuity could have improved by the treatment.

### Questionnaires

The original AS-20 [5] contained two subscales with ten items each: “psychosocial” (items 1–10) and “function” (items 11–20). All items of the original AS-20 have a five-point scale, and all responses converted linearly to a range of 0–100 (worst to best). A Dutch translation of the original version of May 2008 [5] was applied in the study. The original AS-20 was translated, back-translated, and this was evaluated by two native English-speaking ophthalmologists and checked for the semantic consensus between the original AS-20 and the Dutch version. A Rasch analysis of the AS-20 in 2012 showed that each subscale had two subscales, giving four subscales: self-perception, interaction, reading function, general function, with 18 items [13].

The A&SQ has 26 items divided over five subscales (termed “domains”): Fear of losing the better eye (items 1–3), distance estimation (items 4–13), visual disorientation (items 14–16), diplopia (items 17–21), and social contact and cosmetic problems (items 22–26). All 26 A&SQ items had a five-point scale for responses except items 1, 4, 5, that had a two- or three-point scale. For items 8, 13, 14, 15, 16, 18, 25, and 26 the answer “not relevant” was added as response alternative, i.e., not applicable. In Europe, most people drive a car, practice sport, use trains, and go shopping, so these items contribute to vision-specific quality of life.

### Factor analysis

Imputation for missing and non-applicable answers was based on six-component singular value decomposition (SVD). SVD would be the best method to neutralize the effect of missingness on the outcomes, as it is connected with factor analysis, which we wanted to perform next. In this case, the non-applicable response-category denotes missing by design, so there is no dependence of the missingness on what the value would have been, which statistically can be considered as missing at random. Imputation is then allowed, i.e., gives unbiased estimates, if the missing response on an item was for making inferences about the functional status of patients, i.e., quality of life; not allowed when judging the item for its use in an item bank. SVD, a multiple imputation method, starts with the initial imputed value of the mean after which any imputed value is in turn calculated by iteration steps of SVD until stability is achieved in the imputed values. The SVD was applied to impute 266 values (missing and non-applicable answers) out of 5060 values from 110 patients. After imputation, factor analysis was performed on the correlation matrix belonging to the single combined set of data. After computing polychoric correlations to correct for the discreteness of the item responses, it was examined whether and how the two AS-20 subscales and the five A&SQ domains had underlying quality-of-life factors in common. Responses on all items were regressed on a number of unobserved underlying factors, i.e., responses on all items were factor analyzed simultaneously. The regression coefficients of the item responses on these factors (the factor loadings) were estimated such that they could reproduce the sample inter-item correlation matrix of the item responses. Factors across all items were identified by estimating the factor loadings per question and the factor scores per respondent that gave the best fit to the responses on the 25 items of the A&SQ questions (question 1 is excluded from factor analysis as this is a routing question) and the 20 items of the AS-20. The strength of a factor was quantified by the percentage of variance in the responses on all items that was explained by the factor: the higher the percentage, the stronger the factor. Summation of the squared factor loadings across questions resulted in the eigenvalue of a factor. If the eigenvalue of a factor was smaller than 1.0, i.e., a variance smaller than the variance of a single standardized item response, it was assumed that the factor did not contribute to the responses on the combined questionnaires. After structuring the dimensionality of the factor space by the identified number of factors, the factors were rotated orthogonally (varimax rotation) so that clusters of items could be more easily related to factors. We took into account all correlations between the factors and the items, i.e., no factor threshold, as there is no standard rule for applying a threshold for clustering of for instance 0.3.

The identified factors were correlated with past and current clinical variables of strabismus angle, degree of binocular vision, and visual acuity of the worse eye, as in our previous study on the clinical validation of the A&SQ [11].

There is a delay between presenting the two instruments. The A&SQ had been presented to the patients in 2002, whereas the AS-20 was first mentioned in 2007 [4], published in 2009 [5] and presented to our patients in 2009 and 2010. The discussion about the appropriate quality-of-life instruments for strabismus and amblyopia patients only started in 2010 [14]. Thereafter, we decided to resolve this debate by analyzing the combined data set of AS-20 and A&SQ responses in the same patients. This resulted in the delay of 8 years between presenting the A&SQ [3] and the AS-20 to the same persons.

## Results

### Study participants

Of the AS-20 questionnaires that were sent out in 2009–2010, 110 were returned and completed. The mean age of the 110 respondents was 44 years; range, 38–51 years, and 53 (45.4%) were male.

For 93 persons (85%), data about the visual acuity of worse eye, degree of binocular vision and the manifest angle of strabismus were retrieved from their orthoptic re-examination in 2003, in addition to their orthoptic examination at childhood [11, 12]. The diagnosis of the 93 re-examined persons was as follows: 53 had strabismus and amblyopia; 17 had anisohypermetropia and amblyopia; 20 had strabismus, anisohypermetropia and amblyopia and three had deprivation amblyopia. This means that of the 93 persons, 20 did not have strabismus (22%). None had diplopia.

### AS-20 subscale scores

In order to determine the convergent validity of the two AS-20 subscales, psychosocial and function, we calculated the means of total score (*N* = 110) per AS-20 item. Scores ranged from 0 (worst quality of life) to 100 (best quality of life). The total score means per AS-20 item on the psychosocial subscale were: 89, 88, 90, 91, 98, 50, 98, 91, 94, and 95 for items 1 to 10, respectively. The mean of total score on the psychosocial subscale was 88. The means of total score per AS-20 item on the function subscale were 81, 90, 83, 66, 74, 78, 91, 70, 92, and 83 for items 11 to 20, respectively. The mean of total score on the function subscale was 81.

### Factor analysis

Although factor analysis on the correlation matrix found six factors with eigenvalue greater than 1.0, only the first four factors appeared to be of sizeable importance, each accounting for at least 0.10, i.e., 10% of the combined variance (Table 1). Cumulatively, the six factors explained 78% of the variance in the combined set of AS-20 and A&SQ items.

**Table 1:** The factor analysis found six factors that explained 23, 18, 13, 14, 7, and 5% of the variance of the responses on the 45 items. In the table, the factor loadings show to what extent each item loaded on each of the found factors, representing quality-of-life dimensions. The items have been grouped per factor with their maximal loading (bold). If this loading approaches 1.0, the response to the item correlates well with the found factor. For instance, the AS-20 item 14 and A&SQ items 4–19 all have a high loading (0.53–0.81) on factor 2, the quality-of-life dimension related to depth perception. *Ps * psychosocial, *Sc* social contact and cosmetic problems, *Fu* function, *De* distance estimation, *Di* diplopia, *Fe* fear of losing the better eye, *Vd* visual disorientation, *Fc* factor

Item	Subscale and Item description	Fc 1	Fc 2	Fc 3	Fc 4	Fc 5	Fc 6
AS20 1	Ps I worry about what people will think about my eyes	**0.93**	0.1	0.14	0.05	0.01	−0.03
AS20 2	Ps I feel that people are thinking about my eyes	**0.92**	0.08	0.07	0.01	0.04	0.14
AS20 3	Ps I feel uncomfortable when people are looking at me	**0.94**	0.14	0.03	0.07	0.13	0.17
AS20 4	Ps I wonder what people think when they look at me	**0.94**	0.14	−0.01	0.01	0.02	0.11
AS20 7	Ps People avoid looking at me because of my eyes	**0.76**	−0.01	0.27	0.14	0.13	−0.18
AS20 8	Ps I feel inferior to others because of my eyes	**0.71**	0.27	0.21	0.07	0.34	0.12
AS20 9	Ps People react differently to me because of my eyes	**0.78**	0.3	0.07	0.01	0.16	0.22
AS20 10	Ps I find it hard to initiate contact with people I do not know	**0.85**	0.17	−0.04	0.09	0.13	0.11
ASQ 22	Sc I have difficulty with eye contact in personal conversations	**0.64**	0.07	0.07	**0.64**	−0.13	−0.09
ASQ 23	Sc I have difficulty with eye contact in group conversations	**0.71**	0.13	−0.04	**0.52**	−0.12	−0.15
ASQ 24	Sc My eyes are misaligned (eyes cross, turn out or up)	**0.66**	0.21	0.05	0.22	0.29	−0.16
ASQ 25	Sc Because of misaligned eyes I feel insecure	**0.71**	0.16	0.11	0.49	0.03	−0.22
ASQ 26	Sc If I did not have misaligned eyes, I would be more self-confident	**0.68**	−0.06	0.48	0.15	−0.46	−0.02
AS20 14	Fu I have problems with depth perception	0.16	**0.81**	0.24	−0.04	0.09	−0.1
ASQ 4	De I can estimate distances well	0.28	**0.68**	0.31	0.36	0.01	−0.24
ASQ 5	De I have good depth perception	0.24	**0.76**	0.26	0.27	0.01	−0.14
ASQ 6	De I feel unsure when putting something on a table	0.01	**0.79**	−0.09	0.41	0.25	0.22
ASQ 7	De I miss the other person’s hand when shaking hands	−0.03	**0.77**	−0.23	0.3	0.12	0.19
ASQ 8	De I have difficulty parking my car	0.18	**0.53**	0.15	0.03	0.25	**−0.63**
ASQ 9	De I find it difficult to put the cap on a pen or marker	0.11	**0.81**	−0.15	0.42	0.24	0.00
ASQ 10	De I find it difficult to put a power plug into a socket	0.12	**0.77**	−0.13	**0.54**	0.07	0.06
ASQ 11	De I have difficulties pouring drinks	0.21	**0.76**	−0.08	0.3	0.21	−0.01
ASQ 13	De I have difficulties playing ball	**0.51**	**0.77**	−0.16	0.05	0.09	0.02
ASQ 19	Di When I am tired I must be careful not to miss hold	0.18	**0.79**	0.33	0.17	−0.08	0.13
AS20 12	Fu I avoid reading because of my eyes	0.16	0.05	**0.92**	0.08	−0.05	0.02
AS20 13	Fu I stop doing things because my eyes make it difficult to concentrate	0.24	−0.11	**0.83**	−0.09	0.27	0.07
AS20 15	Fu My eyes feel strained	0.02	0.12	**0.72**	0.03	0.14	0.17
AS20 16	Fu I have problems reading because of eye condition	−0.05	−0.03	**0.82**	0.14	0.21	−0.14
AS20 17	Fu I feel stressed because of my eyes	0.45	−0.05	**0.54**	0.17	0.35	0.17
AS20 19	Fu I cannot enjoy my hobbies because of my eyes	0.37	0.19	**0.58**	0.34	0.1	0.48
AS20 20	Fu I need to take frequent breaks when reading because of my eyes	−0.04	0.03	**0.86**	0.14	−0.07	−0.04
ASQ 2	Fe I worry about losing the better eye	0.04	0.17	0.01	**0.85**	0.04	0.22
ASQ 3	Fe I worry something might get into my eye	0.04	0.25	0.12	**0.81**	0.09	0.24
ASQ 12	De I have difficulties walking down stairs	0.06	**0.51**	−0.02	**0.69**	0.16	−0.18
ASQ 14	Vd I have difficulties finding way in a shopping mall	0.18	0.37	0.28	**0.61**	0.27	−0.27
ASQ 15	Vd I have difficulties finding way in a department store	0.18	0.37	0.21	**0.8**	0.13	−0.18
ASQ 16	Vd I have difficulties finding way in a train station	0.09	0.38	0.18	**0.7**	0.19	−0.07
ASQ 18	Di Double vision disturbs me in my daily activities	0.45	0.31	0.31	**0.68**	0.14	0.06
AS20 6	Ps I am self-conscious about my eyes	0.43	0.03	0.22	0.12	**0.59**	0.25
AS20 11	Fu I cover or close one eye to see things better	0.04	0.26	0.23	−0.02	**0.74**	−0.09
AS20 18	Fu I worry about my eyes	0.3	0.06	0.16	0.36	**0.67**	0.28
ASQ 21	Di I have to squint or shut one eye in bright sunlight	0.07	0.25	0.03	0.26	**0.67**	−0.13
AS20 5	Ps People do not give me opportunities because of eyes	0.33	0.11	0.27	−0.06	0.16	**0.63**
ASQ 17	Di I see double	0.27	0.17	0.26	0.2	0.01	0.25
ASQ 20	Di I have to do things more slowly when I am tired because of my eyesight	0.48	0.39	0.43	0.16	−0.07	0.00
	Variance explained	0.23	0.18	0.13	0.14	0.07	0.05
	Cumulative variance explained	0.23	0.40	0.53	0.67	0.74	0.78

**Fig. 1:**
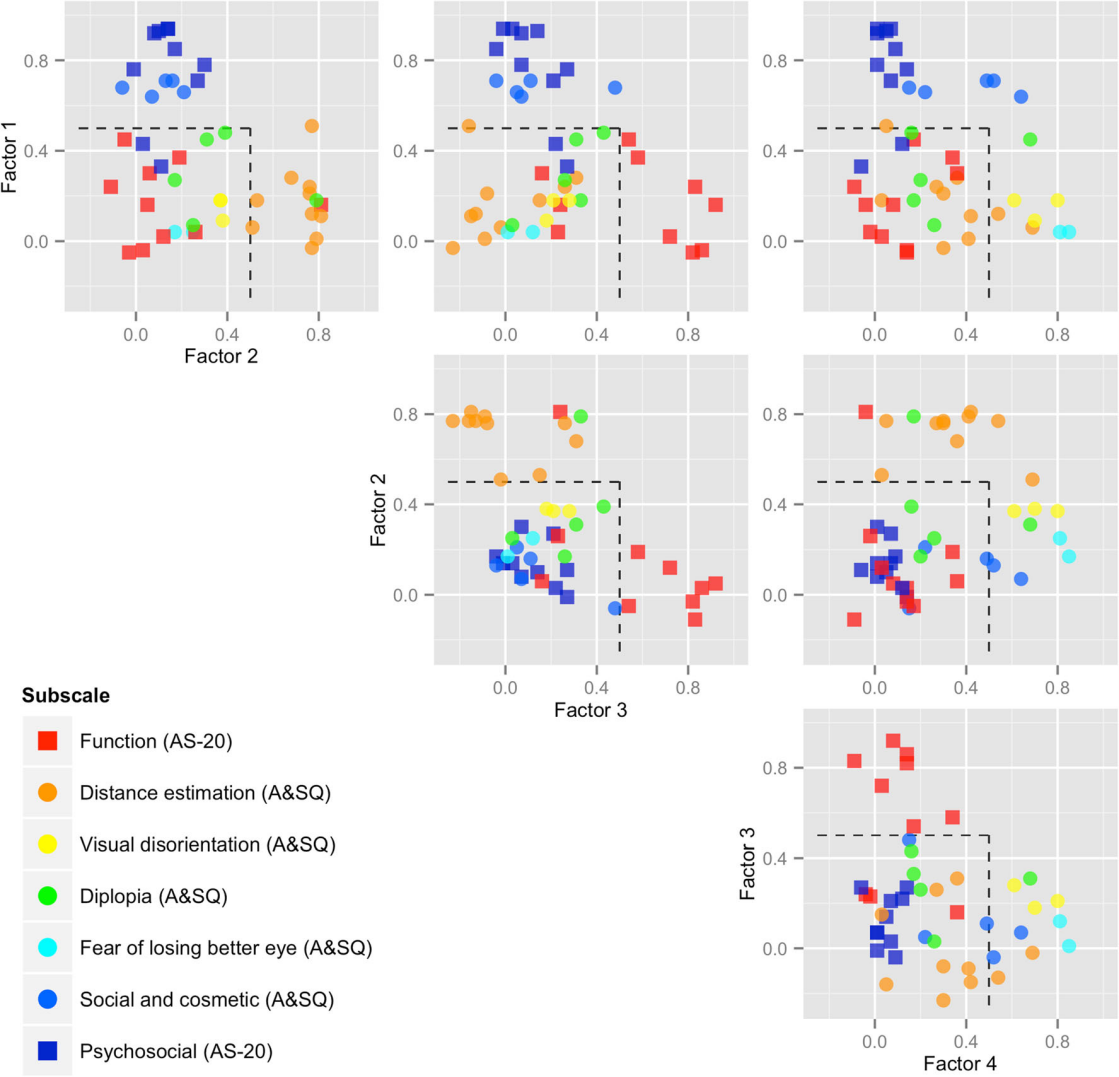
To visualize the factor analysis results presented in Table 1, the factor loadings on the four major factors of the AS-20 and A&SQ combined have been plotted against each other (orthogonally varimax rotated). Throughout the scatterplots of factor loadings given in Fig. 1, *squares* denote AS-20 items and *circles* denote A&SQ items; these have been colored according to their AS-20 and A&SQ subscales. Factor 1 denotes psychosocial, factor 2 depth-perception, factor 3 eye strain, and factor 4 vision and disorientation. For instance, in the *upper left plot*, the loadings of the items of factor 1 are set against the loadings of the items of factor 2. The items that load on factor 1 group together in the left upper quadrant, whereas the items that load on factor 2 group together on the right lower quadrant. In the *upper middle plot*, factor 1 is set against factor 3; in the *upper right plot*, factor 1 against factor 4; in the *middle plot*, factor 2 against factor 3; in the *right plot*, factor 2 against factor 4; and in the *lower plot*, factor 3 against factor 4. The *scattered line* delineates a factor loading of 0.5

Factor 1, explaining 23% of the variance, contained eight of the ten items of the psychosocial AS-20 subscale: items 1, 2, 3, 4, 7, 8, 9, and 10 and by all five items from the A&SQ social contact and cosmetic domain: items 22–26, suggesting a single psychosocial domain in the combined data (see Fig. 1 upper left and upper middle plots for cluster). Two of these items, A&SQ 22 and 23, also loaded on factor 4 (Table 1, Fig. 1 right plot).

Factor 2, explaining 18%, contained 9 items from the distance estimation-domain of the A&SQ: items 4–11 and 13, item 19 from the A&SQ diplopia domain, and item 14 from the AS-20 function subscale; suggesting a domain (See Fig. 1 upper left and middle plots for cluster) in the combined data set that was mostly defined by depth perception items. Two of the items, A&SQ 10 and 12 also loaded on Factor 4. Item 12 of the A&SQ loaded more on Factor 4, hence, assigned to Factor 4 (Fig. 1 upper right plot).

Factor 3, explaining 13%, contained 7 items from the AS-20 function subscale: items 12, 13, 15, 16, 17, 19, and 20 (See Fig. 1 upper middle, middle, and lower plots for cluster) which all pertained to “eye strain”, “concentration” and “reading problems”.

Factor 4, explaining 14%, covered by 7 items from various A&SQ domains: items 2, 3, 12, 14, 15, 16, 18 (See Fig. 1 lower plot for cluster) which all appeared to be vision related. Two more factors with eigenvalue >1, Factor 5 and Factor 6, contained a total of 5 items derived from both questionnaires: from the AS-20 items 5, 6, 11, and 18; and from the A&SQ item 21. Two remaining A&SQ items: items 17 and 20 did not load on any of the six factors.

In the remainder, we describe the five factors with short names that correlate best with the involved items. Hence, factor 1 is named “psychosocial”, factor 2 “depth-perception”, factor 3 “eye strain”, factor 4 “vision and disorientation”, factor 5 “undefined”.

### Correlation with clinical variables

Of the 110 persons, 93 had been re-examined in 2003 and hence, both childhood and recent clinical (orthoptic) parameters could be retrieved of these persons. These were degree of binocular vision [15], manifest angle of strabismus, and Snellen visual acuity of the worse eye. The distribution of the 93 patients over the categories of the visual acuity of the worse eye (decimal values), the degree of binocular vision (ordinal values), and manifest angle of strabismus (categories of strabismus angle) is given in Table 2.

**Table 2:** Three main current clinical variables (of 93 patients out of 110): Visual acuity (linear) of the worse eye. Degree of binocular vision: Bagolini negative, Bagolini positive, Bagolini and Titmus fly positive, Titmus circles 200″-140″ positive, Titmus circles 100″ – 40″ positive, Lang or TNO Plate V positive, TNO Plate VI or VII positive. Angle of strabismus in degrees: negative sign denotes divergent strabismus. Original orthoptic data from childhood was available from all 110 patients

Angle of strabismus, convergent angles are positive	*N* (%)
+5° to +20°	8 (8.6)
0° to +5°	43 (46.2)
0°	30 (32.2)
0° to −5°	7 (7.5)
−5° – −20°	5 (5.4)
Binocular vision	
Bagolini negative	31 (29.2)
Bagolini positive	20 (18.9)
Bagolini and Titmus fly positive	15 (14.1)
Titmus circles 200″-140″ positive	22 (20.8)
Titmus circles 100″-40″ positive	3 (2.8)
Lang or TNO Plate V positive	4 (2.8)
TNO Plate VI or VII	10 (10.6)
Visual acuity of the worse eye (decimal values)	
At least 0.8	47 (44.3)
Less than 0.8, at least 0.5	21 (19.3)
Less than 0.5, at least 0.1	38 (35.8)

The clinical variables of visual acuity of the worse eye and degree of binocular vision regressed significantly on three of the four main factors (Table 3). Acuity of the worse eye regressed significantly on the second factor: items related to depth perception and on the fourth factor: items related to vision. Degree of binocular vision regressed significantly on the first factor: items related to psychosocial and social contact. The diagnosis of the 93 re-examined patients was strabismus and amblyopia (53), anisohypermetropia and amblyopia (17), strabismus, anisohypermetropia and amblyopia (20), and deprivation amblyopia (3). These were not significantly correlated with the AS-20 and A&SQ responses.

**Table 3:** Multiple regression of the five factors on the three clinical variables. Visual acuity of the worse eye correlated most with the items in both questionnaires. Strabismus angle did not correlate significantly with any of the factors (most had been treated adequately by surgery) and binocular vision correlated with psychosocial and social contact items from both questionnaires. *Msq* mean squared, *df2* 2° of freedom, *F* F-statistic. Factor 1 concerned psychosocial problems, factor 2 depth-perception, factor 3 eye strain, factor 4 vision problems, factor 5 undefined problems

	Visual acuity of worse eye				Degree of binocular vision				Strabismus angle			
Factors	Msq.	df2	F	*p* value	Msq	df2	F	*p* value	Msq	df2	F	*p* value
1 Psychosocial	10.505	89	3.20	0.077	50.328	89	15.32	0.000***	9.28	89	2.86	0.096
2 Depth-perception	15.434	89	19.5	0.000***	0.008	89	0.01	0.922	0.014	89	0.18	0.676
3 Eye strain	0.691	89	0.96	0.329	0.479	89	0.67	0.416	0.0341	89	0.05	0.829
4 Vision and disorientation	7.394	89	20.30	0.000***	0.155	89	0.42	0.516	0.020	89	0.50	0.816
5 Undefined	1.122	89	13.95	0.000***	0.096	89	1.19	0.278	0.007	89	0.08	0.773

*p < 0.05, **p < 0.01, ***p < 0.001

## Discussion

Factor analysis suggests that the AS-20 and A&SQ measure a similar psychosocial quality-of-life domain. However, function problems like avoidance of reading, difficulty in concentrating, eye stress, reading problems, inability to enjoy hobbies, and need for frequent breaks when reading are represented only in the AS-20, not in the A&SQ. Their exclusive representation in the AS-20 Function subscale has two causes. First, during the development of the A&SQ, asthenopic items were considered insufficiently specific for strabismus and were excluded a priori. Secondly, during the development of the AS-20, items were generated with adult patients who had, in majority, adulthood-onset strabismus and diplopia (57–62%) [4, 5] and were, hence, more likely to develop such complaints than our adult patients with childhood-onset strabismus and/or amblyopia. The AS-20 was tested in glaucoma and cataract patients to determine its clinically discriminative validity [5], but its specificity has not been tested in patients with, for instance, incorrect glasses, who may well have eye strain, stress, and difficulties with reading and concentrating.

Whether a quality-of-life instrument for strabismus patients should contain such items, which may not be specific for strabismus, is open for debate.

The factor concerning fear of losing the better eye and visual disorientation was only represented in the A&SQ because the A&SQ covers both strabismus and amblyopia.

Depth perception complaints are overrepresented in the A&SQ. The focus group that developed the A&SQ expected that loss of depth perception would play a major role in the decrease in quality of life in amblyopia and/or strabismus patients and, consequently, the A&SQ contains too many items on near and on far distance estimation [16].

Viany et al. [14] and Hatt et al. [4] consider the use of one quality-of-life questionnaire for both amblyopia and strabismus undesirable. They prefer one questionnaire for strabismus and one for amblyopia and to use both questionnaires together in patients with both strabismus and amblyopia. In an evaluative study of the A&SQ, Viany et al. found a dichotomy between amblyopia and strabismus patients by Rasch analysis. That these two groups segregated on a different functioning of the A&SQ items might be explained by the kind of patients that Viany et al. used in their Rasch analysis: their patients mostly had either deep amblyopia or severe strabismus, whereas such patients actually form a minority. Most patients have mild amblyopia after treatment and most patients have mild strabismus after surgery. Many patients have both amblyopia and strabismus.

We think that our use of one questionnaire for strabismus and amblyopia is justified by the fact that strabismus and amblyopia very often occur together. In the RAMSES birth cohort study (*N* = 4624) [17], amblyopia had a prevalence of 3.4%; a third was caused by strabismus and a third by strabismus in combination with anisohypermetropia. Hence, 2% of this large population-based birth cohort had both strabismus and amblyopia, an indication that strabismus and amblyopia occur very often in combination. Our historic cohort, an almost random sample of patients with amblyopia and strabismus, all treated by the only orthoptist in the Waterland area at the time, consisted of patients of whom 56% had strabismus and amblyopia, 22% had strabismus, anisohypermetropia, and amblyopia, and 22% had amblyopia only.

The good performance of the common constructs is demonstrated by the fact that six found factors together explained 78% of the total variance of the combined AS-20 and A&SQ. This is even higher than the 70.5% of the total variance of the A&SQ that was explained by six factors that were found in our previous A&SQ factor analysis [10]. Bian et al. also found six common factors in the A&SQ that explained 67.6% of the total variance [7]. For the AS-20, Hatt et al. found two factors that explained 68.8% of the variance of the AS-20 [5].

As in our previous clinical validation study of the A&SQ by orthoptic re-examination in 2003 [11], we found that visual acuity of the worse eye is correlated most with all factors of both questionnaires. Strabismus angle did not correlate significantly with the factors found, not even with the psychosocial factor. Most of the adult patients with strabismus had been treated adequately by surgery. As in 2003 [11], we again found that binocular vision correlated with psychosocial and social contact items. This correlation might be an effect of impaired binocular vision that most of the adult patients (most of them with microstrabismus or infantile esotropia [11]) had [12]. We have difficulty to offer an explanation about the consequence of impaired stereopsis for low self-image and low self-esteem in the adult patients. Yet, due to impaired stereopsis, initiating eye contact in conversation [18] might be affected by impaired mutual gaze that makes it difficult to locate faces and gazes of other persons and impairs the guidance of making and breaking eye contact [19].

A weakness of our study was the interval between completing the A&SQ in 2002 [3] and the AS-20 in 2010 by the same persons at the age of 36 years and of 44 years, respectively. The AS-20 was published in 2009 [5] while only in 2010 a discussion started about the appropriateness of the two quality-of-life instruments for strabismus and amblyopia.

We acknowledge that several functions may have deteriorated during the span of 8 years. First, presbyopia may have become manifest between the age of 36 years and 44 years and, hence, a patient may have needed reading glasses. The presbyopia may have caused the complaints of avoidance of reading, difficulty in concentrating, eye stress, reading problems, inability to enjoy hobbies, and need for frequent breaks when reading [20]. Such items that may have been caused by presbyopia were mentioned only in the AS-20, which was administered to the patients when they were 44 years old, not in the A&SQ that was administered when they were 36 years old. Secondly, the patient’s visual acuity may have impaired, due to the deterioration of the refractive function of the lens or, unlikely at this age, of the retina. Thirdly, other visual functions may also have been impaired during the interval, like contrast sensitivity, extent of visual field, or color vision. Finally, other physical functions may have been impaired that affect vision-related quality of life.

On the other hand, the few long-term studies about visual acuity, other visual functions, and physical functions affecting vision [21] report that the deterioration occurs more rapidly in elderly patients. Presbyopia may start at the age of 40 years but between 45 and 55 years of age, the deterioration in accommodation occurs most rapidly [22]. Currently, we are considering the possibility of combining the best items of both questionnaires into a new quality-of-life instrument on the basis of the factor analysis of the combined questionnaires.
